# Deep Learning-Based Detection of Bone Tumors around the Knee in X-rays of Children

**DOI:** 10.3390/jcm12185960

**Published:** 2023-09-14

**Authors:** Sebastian Breden, Florian Hinterwimmer, Sarah Consalvo, Jan Neumann, Carolin Knebel, Rüdiger von Eisenhart-Rothe, Rainer H. Burgkart, Ulrich Lenze

**Affiliations:** 1 Department of Orthopedics and Sports Orthopedics, Klinikum rechts der Isar, Technical University of Munich, 81675 Munich, Germany; 2 Institute for AI and Informatics in Medicine, Technical University of Munich, 81675 Munich, Germany; 3 Department of Diagnostic and Interventional Radiology, Klinikum rechts der Isar, Technical University of Munich, 81675 Munich, Germany

**Keywords:** cancer, bone tumor, pediatrics, artificial intelligence, deep learning

## Abstract

Even though tumors in children are rare, they cause the second most deaths under the age of 18 years. More often than in other age groups, underage patients suffer from malignancies of the bones, and these mostly occur in the area around the knee. One problem in the treatment is the early detection of bone tumors, especially on X-rays. The rarity and non-specific clinical symptoms further prolong the time to diagnosis. Nevertheless, an early diagnosis is crucial and can facilitate the treatment and therefore improve the prognosis of affected children. A new approach to evaluating X-ray images using artificial intelligence may facilitate the detection of suspicious lesions and, hence, accelerate the referral to a specialized center. We implemented a Vision Transformer model for image classification of healthy and pathological X-rays. To tackle the limited amount of data, we used a pretrained model and implemented extensive data augmentation. Discrete parameters were described by incidence and percentage ratio and continuous parameters by median, standard deviation and variance. For the evaluation of the model accuracy, sensitivity and specificity were computed. The two-entity classification of the healthy control group and the pathological group resulted in a cross-validated accuracy of 89.1%, a sensitivity of 82.2% and a specificity of 93.2% for test groups. Grad-CAMs were created to ensure the plausibility of the predictions. The proposed approach, using state-of-the-art deep learning methodology to detect bone tumors on knee X-rays of children has achieved very good results. With further improvement of the algorithm, enlargement of the dataset and removal of potential biases, this could become a useful additional tool, especially to support general practitioners for early, accurate and specific diagnosis of bone lesions in young patients.

## 1. Introduction

Unspecific pain around the knee in children is not uncommon, and for it to be caused by a bone tumor is rare [1]. Despite the sparseness of malignancies in children, they cause the second most deaths before the age of eighteen [2]. In this group, bone tumors are more common than later in life and occupy the sixth place of all malignancies [3]. Tumors of bone mostly occur around the knee, with 50% of all osteosarcomas and 30% of all Ewing sarcomas in this region (Phemister’s Law) [4].

One challenge in children with bone tumors is the early detection of the disease [5]. Even patients with highly malignant sarcomas often present initially with non-specific pain [6] which might be misinterpreted as growing pain [7]. However, if symptoms persist over time, the first diagnostic tool are usually conventional X-rays. Most bone tumors can be seen on X-rays, but, especially in the early phase get overlooked regularly, which results in a delayed diagnosis [8].

Even though a delayed diagnosis and treatment initiation does not necessarily seem to influence the prognosis [6,8,9,10,11], the passage of time may nonetheless lead to tumor growth and the development of metastases [8,12]. Both these factors are known predictors for a worse outcome in sarcomas and negatively influence the treatment by complicating the surgical treatment [13,14,15].

Novel and more far-reaching analysis tools for image interpretation are urgently needed, especially for general practitioners, as well as hospitals and clinics with limited experience and resources for the detection and interpretation of rare bone diseases [16]. Deep learning (DL) is a subfield of machine learning and a distinct application of artificial intelligence (AI) that evolved from pattern recognition and learning theory. While complex data analysis of cancer tissues using AI models and imaging data is already widely used in some medical research fields (e.g., lung and breast cancer [17]), the application of these methods in orthopedic oncology is still very limited [16]. The fact that widespread structures for systematic data collection have not yet been established worldwide and that sarcomas are very rare and heterogeneous entities significantly complicates modern AI applications, for which a sufficient and qualitative amount of data is crucial. Although this is a common obstacle—especially in medicine—several techniques have evolved to deal with limited data availability. One popular technique is data augmentation [18], in which new data are artificially generated by applying small transformations to the original data. An even more powerful method is transfer learning [19], in which a model is developed for a source task and then reused as a starting point for the target task.

Research using AI in bone tumor detection and classification has already been published. In 2020, He et al. were able to develop an algorithm that could classify bone tumors in three grades of malignancy [20]. Similarly, our research group developed an algorithm to differentiate between osteomyelitis and Ewing’s sarcoma [21]. In these works, cropped and optimized X-rays were used. Whereas this is a helpful step in the diagnosis of bone tumors, our study focuses on the early detection of these lesions.

The longest delay in diagnostics and treatment takes place before the referral to a specialized center [8,10], which is where our tool could be used. In 2021, Do et al. showed that the detection and classification of bone tumors can be performed using AI in a comparable approach [22]. In this study, however, we focus on children, in which X-rays are more difficult to interpret, because of the growing skeleton [1]. In our clinical experience and backed by research [8], the help in finding tumors is more important than in classifying the specific lesions. After having detected a suspected lesion, patients should be transferred to specialized centers. With our algorithm, we specifically do not want to help find the correct diagnosis before referral to prevent false diagnoses. Instead, we want to help general orthopedic surgeons in finding those patients, who should be transferred for further diagnostics.

Our work aims to facilitate and accelerate the detection of bone cancers around the knee in children by helping to detect suspicious lesions on X-ray images based on state-of-the-art DL methodology.

## 2. Materials and Methods

### 2.1. Eligibility Criteria

A database research of all patients treated (tumor resection) at our institution between 2000 and 2021 was performed. The area around the knee was defined as epiphyses and metaphyses of the long bones and the Patella. In total, 189 patients with histopathologically verified diagnosis of a benign or malignant bone tumor around the knee and age of 18 years or younger were identified. As a control group, we obtained 220 knee X-rays of healthy children. Each patient’s guardian gave written consent for anonymized use of data for research purposes before being enrolled in this study.

The anamnestic and image data were retrieved from our hospital information system (HIS) and the picture archiving and communications system (PACS).

Patients lacking sufficient imaging (no preoperative X-rays) were excluded.

### 2.2. Statistical Analysis

Descriptive data is presented according to the Strengthening the Reporting of Observational Studies in Epidemiology (STROBE) guidelines [23]. Discrete parameters are described by incidence and percentage ratio and continuous parameters by median, standard deviation and variance.

For statistical analysis and evaluation, accuracy, sensitivity and specificity were computed, cross-validated and interpreted by an orthopedic surgeon (S.B.) and a computer scientist (F.H.). The metrics were implemented using the scikit-learn library [24].

A patient with several images was only considered once for statistical analysis.

Age, tumor entity as well as gender were analyzed. None of the patients’ metadata was normally distributed according to the normality test by D’Agostino–Pearson. The correlation matrix according to values of Spearman’s rank-order correlation coefficient, which is a measure for linear correlation between two datasets and does not assume that both datasets are normally distributed, was computed. None of the relations showed a relevant correlation, which is likely with small datasets (‘age/’gender |ρ| = −0.157, ‘age/’entity |ρ| = −0.045, ‘gender/’entity |ρ| = 0.104).

### 2.3. Model Training

Model training and inference were conducted on a DGX Station A100 with four 80 GB graphical processing units (Nvidia Corporation, Santa Clara, CA, USA), 64 2.25 GHz cores and 512 GB DDR4 system memory running on a Linux/Ubuntu 20.04 distribution (Canonical, London, UK). Preprocessing and model implementation were performed in Python 3.11.1 using PyTorch 1.13.1 and Cuda toolkit 12.0. The pretrained model of this study will be provided on GitHub upon publication.

### 2.4. Algorithm

We implemented a pretrained Vision Transformer architecture [25,26]. To cope with the overall limited amount of data and to integrate regularization, extensive data augmentation was implemented to artificially generate more input data during training. To cope with the class imbalance, median frequency balancing was used to weigh the loss of classes appropriately and support the robustness of the algorithm. We used a data split of 60%, 20% and 20% for training, validation and testing, respectively. Because we collected up to three images from individual patients, the data split was applied to patients to avoid cross-contamination and, thus, achieve higher statistical significance. An additional 5-fold cross-validation supported this task, while randomly selected hold-out test data were left untouched for the final analysis.

### 2.5. Plausibility

To provide plausibility and additional insight into the AI model, Gradient-weighted Class Activation Mappings (Grad-CAMs) were implemented in the final inference step [27]. Grad-CAMs utilize the gradient information of a deep learning network to understand specific neurons and their impact on decision making. The result is a colored heat map, which is co-registered with the original input image and indicates where the algorithm found relevant information for the task at hand. This technique was applied to get a better understanding of where the algorithm detects relevant information. To provide a higher statistical significance, the Grad-CAM results were averaged from the 5-fold cross-validation.

**Figure 1:**
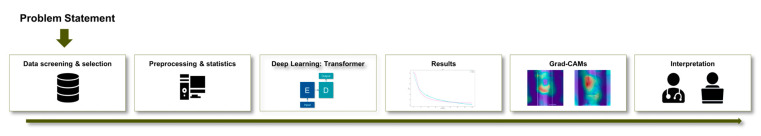
Workflow of the proposed approach (adapted from [22]).

Figure 1 demonstrates the overall workflow of the presented study.

## 3. Results

### 3.1. Dataset

Of the 189 identified patients, 176 children with bone tumors around the knee and a total of 366 images were included in this study (patients lacking preoperative X-rays were excluded). The underlying tumor entities of this group are displayed in Table 1. The median age at diagnosis was 15 years (9 to 18 years), with 56 (32%) females and 120 (68%) males; 76% suffered from benign lesions and 24% from malignant bone tumors. About 10% of the training dataset comprised external data (externally taken X-rays) integrated into our clinical systems from general practitioners, radiologists and pediatricians.

The control group consisted of children, who presented with knee pain or trauma in our outpatient clinic or emergency department. By excluding images with pathologies, a healthy cohort of 220 children was created. The mean age at the time of X-ray imaging was 15. 

### 3.2. Algorithm

All results were cross-validated. The two-entity classification of the healthy control group and the pathological group resulted in an accuracy of 92.2%/89.1%, a sensitivity of 90.9%/82.2%, and a specificity of 97.1%/93.2% for the validation and test groups, respectively.

**Figure 2:**
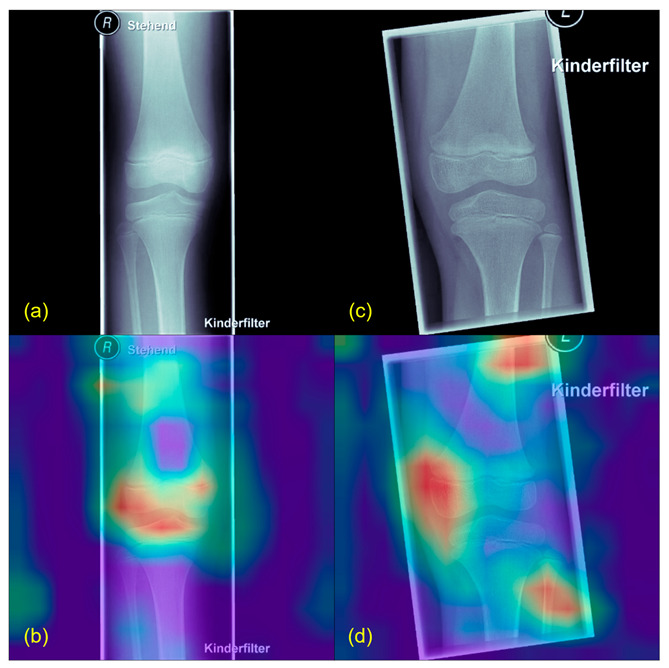
Grad-CAM example from healthy patients. (**a**,**c**) show the original X-rays, (**b**,**d**) the Grad-CAMs of the same images. Highlighted in red are the parts used by the algorithm to find its conclusion. Patients classified as healthy usually showed a more diffuse Grad-CAM.

**Figure 3:**
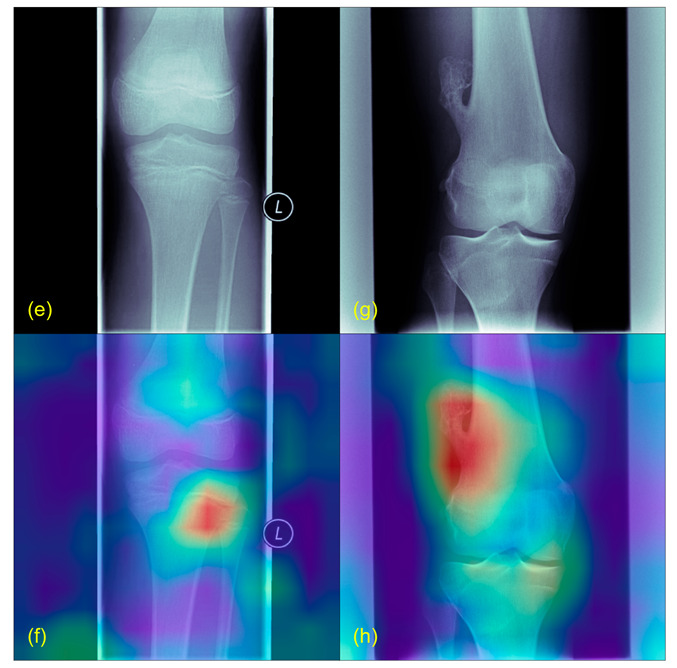
Grad-CAM example with tumors. (**e**) is taken from a patient with an osteosarcoma of the proximal, lateral and left tibia, (**g**) shows an osteochondroma of the distal, lateral and right femur. (**f**,**h**) are the Grad-CAMs of the same images. Highlighted in red are the correctly identified tumors.

**Figure 4:**
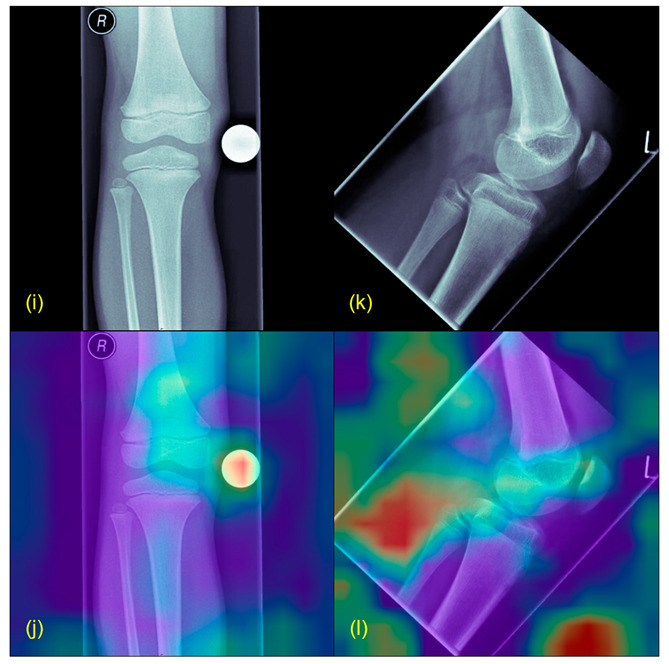
Grad-CAM example from misleading results. (**i**,**k**) show the original X-rays, (**j**,**l**) the Grad-CAMs of the same images. Highlighted in red are the parts wrongly marked as suspicious (for example the scaling ball in (**i**)).

Figure 2 and Figure 3 illustrate exemplary Grad-CAM results from healthy and pathological cases. Figure 4 shows results from misclassified images.

## 4. Discussion

This study aimed at implementing an AI algorithm for facilitating the detection of bone tumors around the knee in X-rays of pediatric patients. The main finding of this study was that our DL model was able to identify pathological knee X-rays by classifying them into suspect and non-suspect classes with very high performance. Furthermore, through Grad-CAMs, we could show that most of the predictions were based on imaging anomalies was a human expert would have classified as suspect, too, when making an assessment and, thus, incorporating elements of explainable AI into our study.

Similar studies have been published, mostly in other medical fields [28]. Studies have shown that the use of AI in the diagnostic work-up of sarcoma can be very helpful [20,21,22]. Whereas digital help in finding the exact diagnosis of bone tumors can certainly facilitate the treatment, this wasnot the aim of this study. The aim of this study was to develop a tool, which can be used by less experienced physicians to detect suspicious bone lesions. From our point of view, the algorithm should not provide a specific diagnosis with regards to a special entity or grade of malignancy, as this could lead to an under-treatment of tumors that are falsely defined as benign.

Our study shares the common goal with the research conducted at Chonnam National University Hospital (CNUH) by Do et al. [22] of utilizing deep learning to detect and classify knee bone tumors in pediatric patients. While both studies aim to assist healthcare providers in radiographic interpretation, there are several distinctions worth noting. Our approach employed a pretrained Vision Transformer architecture with techniques such as median frequency balancing and extensive data augmentation, aiming for a highly sensitive tool that could detect suspected lesions. In contrast, the CNUH study proposed a Seg-Unet model with global and patch-based approaches, focusing on classification, tumor segmentation, and high-risk region segmentation. This allowed them to achieve a high accuracy of 99.05% for classification and a Mean IoU of 84.84% for segmentation. While our study also obtained promising results, our emphasis was on creating an accessible tool for less experienced orthopedic surgeons, potentially through a smartphone application. The CNUH study’s focus on a more specific classification and segmentation method—resulting in higher accuracy—demonstrates the potential breadth of approaches in this critical area of medical imaging, and further underscores the need for collaboration and exploration of various methods to enhance early detection and treatment of bone tumors.

Another similar study by He et al. [20] focused on developing a deep learning model to classify primary bone tumors from preoperative radiographs and offers a distinct approach compared to our study, emphasizing multi-institutional collaboration and a comparison with radiologists’ performance. While our work aimed at identifying suspected lesions to refer patients to specialized centers, their research went a step further in differentiating benign, intermediate, and malignant tumors, achieving an AUC of 0.894 and 0.877 for benign vs. non-benign on cross-validation and external testing, respectively. They even compared the model’s performance with radiologists, showing similar accuracy to subspecialists. In contrast, our study centered on the implementation of a pretrained Vision Transformer architecture, with the added dimension of explainable AI through Grad-CAMs, and an intention to develop a tool possibly accessible through a smartphone application. While both studies recognize the importance of deep learning in bone tumor detection, their focus on a more refined classification and direct comparison with human experts brings an additional layer of validation, emphasizing the complementary roles of AI and human expertise in medical diagnosis.

The proposed algorithm should be a highly sensitive tool for the lesser experienced orthopedic surgeons to detect which patients should be referred to specialized centers, where the diagnostic run-up and treatment can be completed.

One limitation of this study is that—until now—our model can identify suspicious X-ray images as a whole but not clearly detect where the potential tumor is. Although this could already be a helpful tool for early bone tumor detection and a decrease of “time to diagnosis”, we intend to optimize our algorithm with respect to, e.g., incorporating clinical data as well as detecting the tumor contours precisely for a comprehensive tumor assessment using X-rays.

The Grad-CAM results of correctly classified images displayed that the model has focused on pixel areas with real tumor formations or typical predilection sites of bone tumors. In contrast, the Grad-CAM results of misclassified images highlighted background images or, e.g., confounders, such as artifacts or X-ray calibration balls. Confounders present a common issue in image interpretation with deep learning and need to be addressed in future studies.

One further aim of this study was to create a basic platform for the future development of a smartphone application, thus giving less experienced healthcare providers a helpful tool for the bone tumor screening of pediatric X-rays. However, we are aware that the expertise of tertiary centers and human assessment cannot be replaced by AI so far. 

The proposed approach of using modern image interpretation algorithms for pathology detection can be adapted to other scenarios. Furthermore, we applied several methods to provide robustness and generalizability of our predictions (data augmentation, cross-validation, etc.). However, due to the limited data, the dataset might—until now—still inherit unidentified patient biases or confounding biases as shown in Figure 4. These issues need to be addressed in future studies.

X-ray imaging is a common and widely available imaging technique for initial radiological assessment. Although X-ray images consist of only two-dimensional grayscale information, their high resolution and highly standardized technique make them a very suitable input for advanced algorithms. We believe that AI algorithms can be valuable real-time support for any general practitioner involved in the crucial processes of detecting potential bone lesions. This allows for minimal time loss between occurrence of symptoms, initial diagnosis and treatment initiation, which is absolutely critical for patients with malignant lesions. In conclusion, our approach demonstrated very good results with state-of-the-art deep learning methodology. With further improvements such as an increase in the dataset and elimination of potential biases, this algorithm could become an indispensable support tool for the assessment and early detection of bone lesions in young patients.

## Figures and Tables

**Table 1:** Entity distribution.

Entity	Number	Percentage
Ewing’s sarcoma	1	0.6%
Chondrosarcoma	3	1.7%
Giant cell tumor	3	1.7%
Enchondroma	5	2.8%
Chondroblastoma	10	5.7%
Non-ossifying Fibroma	25	14.2%
Osteosarcoma	41	23.3%
Osteochondroma	88	50.0%
Total	176	100.00%

## Data Availability

The pretrained model of this study will be provided on GitHub upon publication.
